# Contributions of Hypoxia-Awareness Training to the Familiarization of Personal Symptoms for Occupational Safety in the Flight Environment

**DOI:** 10.3390/ijerph18062904

**Published:** 2021-03-12

**Authors:** Kwo-Tsao Chiang, Min-Yu Tu, Chao-Chien Cheng, Hsin-Hui Chen, Wun-Wei Huang, Yu-Lung Chiu, Yun-Yi Wang, Chung-Yu Lai

**Affiliations:** 1 Aviation Physiology Research Laboratory, Kaohsiung Armed Forces General Hospital Gangshan Branch, Kaohsiung City 820, Taiwan; charco66@gmail.com (K.-T.C.); du0807@yahoo.com.tw (M.-Y.T.); superbe28@gmail.com (C.-C.C.); huang.wen.wei0@gmail.com (W.-W.H.); 2 School of Public Health, National Defense Medical Center, Taipei City 114, Taiwan; long_ruth0624@mail.ndmctsgh.edu.tw; 3 Department of Health Business Administration, Meiho University, Pingtung County 912, Taiwan; 4 Department of Life Sciences and PhD Program in Translational Medicine, National Chung Hsing University, Taichung City 402, Taiwan; 5 Institute of Medical Science and Technology, National Sun Yat-sen University, Kaohsiung City 804, Taiwan; 6 Department of General Medicine, Tri-Service General Hospital, National Defense Medical Center, Taipei City 114, Taiwan; orchid1319@gmail.com; 7 Graduate Institute of Life Sciences, National Defense Medical Center, Taipei City 114, Taiwan; 8 Emergency Room, Kaohsiung Armed Force General Hospital, Kaohsiung City 802, Taiwan; sccgmaw@gmail.com; 9 School of Nursing, Kaohsiung Medical University, Kaohsiung City 807, Taiwan; 10 Graduate Institute of Aerospace and Undersea Medicine, National Defense Medical Center, Taipei City 114, Taiwan

**Keywords:** hypoxia, aviation medicine, hypoxia-awareness training, altitude chamber

## Abstract

Hypoxia remains a flight-safety issue in terms of aviation medicine. Hypoxia-awareness training has been used to help aircrew members recognize personal hypoxia symptoms. There is still no study, as yet, to establish the association of within-subject data between inflight hypoxia events and the altitude chamber. The main purpose of our study was to use paired subjects’ data on inflight hypoxia symptoms compared with those experienced during training. A questionnaire was developed to obtain information on military aircrew members in 2018. Among 341 subjects, 46 (13.49%) suffered from inflight hypoxia. The majority of the subjects detected ongoing inflight hypoxia on the basis of their previous experience with personal hypoxia symptoms or sensations in previous chamber flights. Of the top five hypoxia symptoms, the data revealed that hot flashes, poor concentration, and impaired cognitive function appeared both during the inflight events and during the hypoxia-awareness training. The occurrence rate of hypoxia symptoms was found to not be significantly different between the in-flight events and the past chamber flights through an analysis of within-subject data. Because the individual memory had faded away over time, fresher hypoxia awareness training is still mandatory and valuable to recall personal hypoxia experience for military aircrew members.

## 1. Introduction

Based on Dolton’s law, atmospheric pressure at sea level is 760 mmHg, but it decreases with increasing altitude. Moreover, oxygen partial pressure decreases and leads to fewer oxygen molecules being inhaled and less air exchanged by human lungs. In this situation, there is insufficient oxygen being delivered to vital organs, thus impairing those functions, especially those related to the brain and nervous system. In flight, physiological altitude is generally defined as 10,000 feet, at which pilots who are occupationally exposed to such an environment have no significant degraded performance [1]. In the early days of aviation, however, the pioneers flew hot air balloons, and two French scientists, Croce-Spinelli and Sivel, in their attempt to reach 25,000 feet, were the first hypoxia victims due to a high-altitude environment [2].

Since then, supplemental oxygen and cabin-pressurization systems have been specially developed to prevent inflight hypoxia. However, fatal hypoxia incidents occasionally occur, which warns all pilots that the hazard of hypoxia to flight safety is always present [3]. On 25 October 1999, a Learjet 35, a small fixed-wing aircraft, was on schedule during a flight from Orlando, FL, USA, to Dallas, TX, USA. However, this plane tragically crashed into the ground, killing both pilots and all the passengers. After the investigation, the significant contributor to this serious accident was deemed hypoxia due to the loss of cabin pressurization [4]. In another case, Helios Airways Flight 522 was flying from Larnaka, Cyprus, to Prague, Czech Republic, on 14 August 2005. Cabin pressurization failure rendered all the aircrew members and passengers unconscious. Thus, there was no one conscious to fly the aircraft, and it ran out of fuel and catastrophically crashed into the terrain below [5]. From the above two cases, we can see that the aircrew members were unaware that they were being exposed to a hypoxic inflight environment and increasingly lost their ability to take the proper emergency actions.

In World War II, an altitude chamber was constructed to enhance hypoxic awareness and allow military pilots to remember their personal symptoms during hypoxia-awareness training [6]. Island and Fraley (1993) analyzed inflight hypoxia events from 1976 to 1990 in the United States Air Force (USAF) [7]. If the aircrew members had no experience with hypobaric-awareness training, then there was an extremely high possibility of them losing consciousness during exposure to a hypoxic environment. According to other studies, pilots could recognize ongoing inflight hypoxia events based on their familiarity with their own personal symptoms and experience during altitude-chamber flights [6,8]. In addition, several studies have reported a consistency in hypoxia symptoms between different sessions of chamber training. The main symptoms during hypoxia-awareness training were impaired mentation, visual changes, dizziness/lightheadedness, paresthesia, tingling, and slurred speech [9,10,11,12]. Currently, refresher training courses are mandatory in many countries (e.g., every three years in Saudi Arabia, and every five years in New Zealand and the United States) [13].

To the best of our knowledge, few studies have explored the characteristics of inflight hypoxia events and investigated their relationship to individual-level symptoms of hypoxia-awareness training [8,14]. Therefore, the aim of this survey was to describe the details of the inflight hypoxia events and examine the within-subject consistency of the inflight hypoxia symptoms with those of hypoxia-awareness training. These findings will be useful for enhancing the benefits of hypoxia-awareness training, and in terms of amendments to future programs.

## 2. Materials and Methods

### 2.1. Study Design

In Taiwan, the Aviation Physiology Research Laboratory (APRL) organizes and executes an aviation physiology training program for military aircrew members. Prior to flight training, aircrew members must complete an initial altitude chamber flight, and then undertake a refresher training course every four years.

A chamber provides a simulated high-altitude environment that emphasizes the effects of changes in atmospheric pressure on the human body (e.g., sinus and ear blockage, tooth pain, and gastrointestinal discomfort). In addition, the other purpose of altitude-chamber training is to recognize the individual-specific hypoxia symptoms of aircrew members at simulated training altitudes.

We conducted this retrospective and noninvasive/experimental survey to collect information related to the inflight hypoxia experience and hypoxia symptoms from previous chamber flights. This research was assessed as low-risk work and approved by the Institutional Review Board of Kaohsiung Armed Forces General Hospital in Kaohsiung City, Taiwan (No. KAFGH 107-02).

### 2.2. Study Subjects

Study subjects were recruited from all the triservice military aircrews that had experience with altitude-chamber flights. We encouraged and invited them to join this research from 1 January to 31 December 2018, when they received the refresher training course at the APRL. Prior to the prechamber flight briefing, the aviation physiologist/instructor obtained informed consent from all the trainees and delivered the questionnaires to collect data on the inflight hypoxia conditions they experienced and their recall memory of the hypoxia symptoms they experienced during previous chamber flights. In total, 341 subjects voluntarily participated in this study. Among them, 46 (13.49%) had experienced an inflight hypoxia problem at some point during their flight career, and were finally enrolled in the data analysis.

### 2.3. Data Collection

We designed an anonymous and structured questionnaire to collect information about the inflight hypoxia events and previous hypoxia experience during the chamber flights. This questionnaire was composed of several parts: Part I, demographic items about the subjects’ personal characteristics (age, gender, station, airframe, flight years, and flight hours); Part II, experience with inflight hypoxia (yes or no) and the characteristics of inflight hypoxia episodes, including the cabin/flight altitude at which these episodes occurred, potential causes of in-flight hypoxia events, and how to detect such events in the future and immediately take corrective measures obtained by the close type options; and Part III, memory of frequently described hypoxia symptoms recognized during the inflight hypoxia incidents and past chamber flights, such as poor concentration, hot flashes, impaired cognitive function, dizziness/lightheadedness, visual disturbances, numbness, air hunger, paresthesia, fatigue, anxiety, tingling, and nausea [15,16,17].

### 2.4. Statistical Analyses

The frequency and proportion of the descriptive statistics were applied to characterize the distribution of all the questionnaire’s data in this survey. Using the McNemar test, hypoxia symptoms that occurred during the inflight hypoxia incidents were then compared with those during the past chamber flights for the eligible participants. All data from this survey were entered and handled using the SPSS 24 program (IBM, Armonk, NY, USA). The significance level was determined by a two-tailed *p* value < 0.05.

## 3. Results

We received 46 questionnaire responses about the inflight hypoxia cases during the study period. The distribution of age subgroups, <30, 30–39, and ≥40 years, were 17.4%, 43.5%, and 39.1%, respectively; only one case involved a female pilot. Of the participants, over 90% were pilots. Fighter pilots (60.9%) most commonly encounter hypoxic conditions, followed by cargo/transporter pilots (26.1%). Moreover, 30.4%, 54.3%, and 15.3% of the participants had <10, 10–19, and ≥20 years of flight experience, respectively. The flight hours of nearly half of all participants totaled more than 2000 (Table 1).

Table 2 summarizes the data obtained in relation to the inflight hypoxia events. The two most common causes of the hypoxia events occurred at altitudes of 15,000–19,999 (37.0%) and 25,000–29,999 (19.6%) feet. Approximately 40% of the inflight hypoxia events were due to abnormal cabin pressurization: depressurization (17.4%) or failure to pressurize (21.7%). There were 13 participants (28.2%) who stated that mask leakage/unsealing and hose disconnection may have triggered the hypoxia events they experienced. There were 10 respondents that reported the reasons for such events were physiological. Five respondents (11.0%) did not explain the causes for such events. The majority of the respondents reported similar hypoxia symptoms and feelings relative to those they experienced during their previous chamber flights. One-fifth of the respondents recognized hypoxia occurrence when conducting oxygen-system checks. Over 80% described that there was mild (58.7%) and significant degradation (21.7%) of flight performance due to the hypoxia impact. They immediately followed emergency oxygen usage (69.2%), descent (69.2%), and even mission-abortion (34.8%) procedures to prevent loss of consciousness.

We calculated the prevalence of the inflight and chamber-flight hypoxia symptoms (Table 3). The majority of aircrew members experienced several hypoxia symptoms. In flight, the aircrew members frequently detected the top five hypoxia symptoms—hot flashes (43.5%), poor concentration (34.8%), impaired cognitive function (32.6%), paresthesia (28.3%), and fatigue (26.1%). In chamber flights, hot flashes, poor concentration, and impaired cognitive function repeatedly appeared during hypoxia-awareness training. Of these symptoms, the most common symptoms that occurred both in flight and during the chamber flights were poor concentration, hot flashes, impaired cognitive function, paresthesia, and visual disturbance, in descending order. We also found that there was no significant difference in the frequency of the in-flight hypoxia symptoms when compared to those during the chamber flights (all *p* values > 0.05).

## 4. Discussion

From this survey, we discovered 46 inflight hypoxia events (13.49%) from the military aircrew members who attended refresher altitude-chamber training in 2018. Hypoxia is still a hazard that threatens flight safety, and the main cause of hypoxia events is a pressurization-system malfunction. Most events were detected by the participants because of their experience with personal hypoxia symptoms or those that they experienced during previous chamber flights. There was a high similarity in terms of symptoms between the inflight hypoxia events and those during the hypoxia-awareness training.

The previous study described that there was a smaller proportion of the inflight hypoxia incidents in the population of student pilots [14]. Simply stated, this study showed that fewer hypoxia cases occurred in aircrew members who were young, had fewer flight years, or had fewer flight hours. The explanation for this may be that when compared with experienced pilots, the junior pilots usually fly during monitored training courses and at relatively low altitudes due to the type of aircraft. These findings noted that the majority of events occurred for the fighter pilots, consistent with the data analyzed by Deussing et al. (2011) [18]. Because there were relatively small samples in this study, we conservatively explained the tendency related to the flight experience. Future extended work will be conducted to investigate the relationship between the inflight hypoxia characteristics and the demographic factors by collecting more cases.

The current data have shown that hypoxia events often occur at altitudes from 15,000 to 19,999 feet, similar to previous data [8,14]. One study used cabin altitude to present the environment in which the pilots actually stayed at that time of inflight hypoxia attacks [6,8]. Because almost all of our respondents did not focus on cabin altitude during such episodes, we were not able to provide an outcome related to altitude in this study. During the period from 1970 to 1980, 298 inflight hypoxia events were recorded by the USAF. Cabin or cockpit decompression (19.46%) was found to be the leading cause of inflight hypoxia, followed by regulator malfunction (15.77%), oxygen-hose disconnection (9.40%), and poor mask fit (4.03%). Our survey also demonstrated that nearly 40% of incidents were attributed to pressurization-system problems, and 20% of them were induced by oxygen-hose disconnection. Interestingly, some participants reported that physiological issues hindered them and were related to hypoxia, such as fatigue, nutrition, and smoking. These self-imposed stressors may increase pilots’ physiological altitude and make them more susceptible to hypoxic environments in flight [19]. These issues are worth assessing in future studies to determine how they affect the hypoxia tolerance of aircrew members. Oxygen-hose disconnection was another important cause of inflight hypoxia conditions. In this study, the results revealed that the majority of cases occurred for fighter pilots. These pilots usually perform acrobatic combat maneuvers and are exposed to a high G environment. The potential explanation for this majority is that the oxygen hose can be disconnected after a high G load if it was not assembled properly before the flight.

In the USAF, an analysis showed that there were 656 inflight hypoxia incidents that involved 606 altitude-chamber-trained and 50 untrained aircrew members. Only 3.8% of those trained experienced a loss of consciousness; however, 94% of the untrained members suffered from a loss of consciousness [20]. A chamber provides a simulated high-altitude environment at ground level. There are several purposes for hypobaric training: (1) to realize the physiological effects of changes in atmospheric pressure on the body, (2) to recognize personal hypoxia symptoms and behavior affected by hypoxia, and (3) to learn the proper corrective techniques using onboard oxygen systems. The abovementioned USAF survey also indicated that nearly one-fourth of aircrew members detected inflight hypoxia on the basis of their experience during chamber flights [7]. Several studies have also shown that aircrew members use their personal hypoxia symptoms from hypoxia awareness training to recognize inflight hypoxia events [8,14,18]. Corresponding to the previous findings, two-thirds of the respondents in the current study also detected hypoxia attacks through their memory or experience during past altitude-chamber training. However, some aircrew members found ongoing inflight hypoxia when they inspected the oxygen system during the flight. The reason for this may be that they were always educated to follow the rules when inspecting the oxygen system in a disciplined manner so as to avoid hypoxia incidents before and during the flight. This check technique is always mentioned during the chamber flight training. Therefore, the aircrew members are regularly accustomed to performing inflight oxygen-system checks and then possibly detecting and correcting hypoxia occurrence in the early flight stage, even without any symptoms. Few studies have revealed data on the behavioral changes affected by these inflight hypoxia conditions. We extended this work to collect information on flight-performance degradation among the participants. More than 20% of the participants described the significant impact of a hypoxia attack on their performance. When exposed to an environment with a low oxygen concentration, humans have a short period of time, called the time of useful consciousness (TUC), to make life-saving decisions. All of the study participants still had the ability to carry out reactive procedures during the TUC. As aforementioned, the military aircrew members could discover and handle inflight hypoxia events based on personal symptoms and sensations from their experience during chamber flights. Therefore, our findings also enhance the importance of hypoxia-awareness training inside altitude chambers.

From the literature review, it can be seen that the results demonstrated that there were variable orders and frequencies of hypoxia symptoms during the chamber flights. Nonetheless, the dominant hypoxia symptoms included mental impairment, coordination issues/slowing response, visual changes, lightheadedness/dizziness, and hot flashes [9,11]. Consistent with former findings, the top five symptoms of our survey by their order of frequency were poor concentration, hot flashes, visual disturbance, impaired cognition, and dizziness/lightheadedness inside the chamber. In terms of the inflight hypoxia events, the most common symptoms reported were paresthesia, lightheadedness, dizziness, decreased mental ability, and hot/cold flashes [8,18]. Our findings present the highly overlapping symptoms of the inflight hypoxia episodes with those of other studies. Although the majority of the main hypoxia symptoms were similar to those of the chamber flights, there was still a mild difference between them, which can be related to the characteristics of multitasking flight duty for the pilots, in contrast to the fact that they merely focus on experienced hypoxia symptoms inside the chamber.

A significant number of military aviators have stated that they recognized their own symptoms and that they were becoming hypoxic and took timely corrective steps because they recalled their altitude-chamber training [8,14]. In terms of the United States Navy, Deussing et al. (2011) described that the overall symptom spectrum of inflight hypoxia was consistent with that of the hypoxia-awareness training [18]. A comparison of the occurrence of “every single” hypoxia symptom between the inflight and chamber-flight conditions has still not been addressed. Of the 16 symptoms in this study, only five illustrated a significant difference in reporting the scenarios by a comparison of group analyses, but included three main symptoms: tingling, difficulty concentrating, and air hunger. In the current study, the method of individual analysis was substituted to manage the within-subject data of every single hypoxia symptom from the two different exposure events. Surprisingly, dominant hypoxia symptoms reappeared in the different patterns, and all the frequencies were not significantly different between the inflight hypoxia and that during the chamber-flight training. Previous studies have identified that the memory of hypoxia symptoms from chamber flights gradually fades away over time [12]; however, the proportion of primary symptoms was not different between the repetitive exposures, especially for senior aircrew members. In other words, hypoxia-symptom memories can be recalled and enhanced by repetitive training. Similarly, nearly 80% of the participants that recalled hypoxia memories from previous trainings had more flight years and experiences with hypoxia-awareness training, which can likely be explained by the consistency of the symptoms between the inflight hypoxia events and the chamber-flight training. Hypoxia-awareness training is properly repeated in fixed time intervals of three to six years, suitable to refresh the memory of hypoxia symptoms, correct use of emergency equipment, and recovery procedures [9,13]. Similar to the former reports, our findings emphasize the necessity of periodic altitude-chamber indoctrination for all aircrew members.

There are still inherent limitations in the current study. First, the underestimation of inflight hypoxia events may exist, because the pilots are more sensitive to this safety issue. Second, we designed a structured questionnaire to obtain the details of hypoxia attacks described by the participants. Because memory can diminish after several years, reporting errors cannot be ruled out from this study. In the future, monitoring devices integrated with the flight or hypoxia-awareness training would be developed to record objective physiological data and reduce the nondifferential errors. Third, some symptoms of physiological events, such as hyperventilation, are very similar to those of a hypoxic condition. Aircrew members probably misdiagnose this event as being a hypoxia case. However, the subjects declared the inflight hypoxia symptoms corresponded to their experiences inside the altitude chamber. They complied with the disciplines and recovered from the event by using oxygen. Thus, misclassification of other events was unlikely to exist in this study. Next, we sampled the participants during the physiological refresher training over one year. Thus, these results are limited and should not be generalized to the whole community of military aircrew members. Finally, a reduced-oxygen breathing device (ROBD) was used to execute hypoxia-awareness training. The hypoxia symptoms induced by this ROBD are in line with those in the altitude chambers [21,22]. Because there is no training capability in our country, we cannot compare the inflight hypoxia data with the ROBD information.

## 5. Conclusions

In summary, we have illustrated that the malfunction of the pressurization system was the main cause of inflight hypoxia events among military aircrew members. Meanwhile, they managed the ongoing inflight hypoxia that relied on the experience of the chamber training. The dominant hypoxia symptoms inside the chamber were poor concentration, hot flashes, visual disturbance, impaired cognition, and dizziness/lightheadedness, in that order. Our analysis emphasized the within-subject reappearance and consistency of considerable hypoxia symptoms during the inflight hypoxia events and those during the chamber flights. Therefore, hypoxia-awareness training plays an important role in acquainting aircrew members with personal hypoxia symptoms and training them how to respond when facing hypoxic situations, contributing to enhanced flight safety.

## Figures and Tables

**Table 1:** Characteristics of study participants.

Variables	*n* (%)
Age (years)	
<30	8 (17.4)
30–39	20 (43.5)
≥40	18 (39.1)
Gender	
Male	45 (97.8)
Female	1 (2.2)
Station	
Pilot	42 (91.3)
Nonpilot	4 (8.7)
Airframe	
Fighter	28 (60.9)
Jet trainer	6 (13.0)
Cargo/transporter	12 (26.1)
Flight years	
<10	14 (30.4)
10–19	25 (54.3)
≥20	7 (15.3)
Flight hours	
<1000	10 (21.8)
1000–1999	14 (30.4)
≥2000	22 (47.8)

**Table 2:** Characteristics of inflight hypoxia events.

Variables	*n* (%)
Altitude (feet)	
<15,000	8 (17.4)
15,000–19,999	17 (37.0)
20,000–24,999	7 (15.2)
25,000–29,999	9 (19.6)
≥30,000	5 (10.8)
Causes	
Depressurization	8 (17.4)
Failure to pressurize	10 (21.7)
Mask leakage/unsealing	3 (6.5)
Hose disconnection	10 (21.7)
Physiology	10 (21.7)
No response	5 (11.0)
Detection	
Personal hypoxia symptoms	16 (34.8)
Sensations like those experienced in previous chamber flights	14 (30.4)
Abnormal sensation	9 (19.6)
Oxygen system check	10 (21.7)
Impact of flight performance	
None	9 (19.6)
Mild degradation	27 (58.7)
Significant degradation	10 (21.7)
Procedures	
Oxygen usage	32 (69.2)
Descent	32 (69.2)
Abortion	16 (34.8)

**Table 3:** Comparison of inflight and chamber-flight hypoxia symptoms.

Symptoms	In Flight *n* (%)	Chamber Flight *n* (%)	Both *n* (%)	*p* Value
Hot flashes	20 (43.5)	22 (47.8)	13 (28.3)	0.804
Poor concentration	16 (34.8)	23 (50.0)	13 (28.3)	0.092
Impaired cognitive function	15 (32.6)	17 (37.0)	8 (17.4)	0.804
Paresthesia	13 (28.3)	14 (30.4)	8 (17.4)	1.000
Fatigue	12 (26.1)	11 (23.9)	4 (8.7)	1.000
Visual disturbance	11 (23.9)	20 (43.5)	6 (13.0)	0.064
Air hunger	10 (21.7)	14 (30.4)	5 (10.9)	0.424
Dizziness/lightheadedness	6 (13.0)	18 (39.1)	4 (8.7)	0.250
Tingling	6 (13.0)	7 (15.2)	3 (6.5)	1.000
Numbness	5 (10.9)	10 (21.7)	2 (4.3)	0.227
Anxiety	4 (8.7)	10 (21.7)	3 (6.5)	0.070
Nausea	0 (0.0)	3 (6.5)	0 (0.0)	-

## Data Availability

The data in this study are collected and owned by the Aviation Physiology Research Laboratory, Taiwan and cannot be shared publicly due to the regulations.
